# Brugada-Type Electrocardiographic Changes Induced by Antipsychotics in a Patient with Resistant Schizophrenia: A Case Report

**DOI:** 10.1192/j.eurpsy.2026.11501

**Published:** 2026-07-31

**Authors:** O. Seyar, A. Khallouk, L. Azizi, S. Belbachir, A. Ouanass

**Affiliations:** Psychiatry, Arrazi university hospital, Salé, Morocco

## Abstract

**Introduction:**

Brugada syndrome is an inherited cardiac channelopathy associated with an increased risk of sudden cardiac death. Certain psychotropic medications, particularly antipsychotics, have been implicated in unmasking Brugada-type electrocardiographic (ECG) patterns. Recognizing such drug-induced cardiac events in patients with severe psychiatric disorders is critical to prevent fatal outcomes.

**Objectives:**

To report a case of Brugada-type ECG changes in a patient with resistant schizophrenia under antipsychotic polytherapy, and to discuss the clinical implications for psychiatric management.

**Methods:**

We describe the clinical course of a male patient with resistant schizophrenia, hospitalized in the Department of Psychiatry for suicidal behavior. His treatment regimen included amisulpride, aripiprazole, and chlorpromazine. Routine investigations were conducted, including ECG, laboratory analyses, and EEG. A cardiology consultation was obtained.

**Results:**

During hospitalization, ECG demonstrated transient coved-type ST-segment elevation in the right precordial leads, consistent with a Brugada-type pattern. Laboratory tests and EEG were unremarkable. Cardiology evaluation confirmed suspicion of Brugada syndrome. Consequently, clozapine initiation was withheld, chlorpromazine was discontinued, and the patient was maintained on amisulpride combined with aripiprazole. He was subsequently referred to a specialized rhythmology unit. ECG abnormalities resolved following the withdrawal of chlorpromazine.

This case highlights the potential of antipsychotic agents, particularly phenothiazines such as chlorpromazine, to induce Brugada-type ECG changes. The mechanisms may involve sodium channel blockade, which is particularly concerning in patients at risk for arrhythmogenic events. Clinicians should be vigilant in monitoring cardiac function when prescribing antipsychotics, especially in polytherapy, and collaborate with cardiology to optimize patient safety.

**Conclusions:**

Brugada-type ECG changes can occur in patients treated with antipsychotics, posing a risk of severe arrhythmia and sudden cardiac death. Careful cardiac monitoring, timely recognition of ECG abnormalities, and interdisciplinary management are essential in such cases.

**Disclosure of Interest:**

None Declared

